# An exploratory study into the effects of extraordinary nature on emotions, mood, and prosociality

**DOI:** 10.3389/fpsyg.2014.01577

**Published:** 2015-01-28

**Authors:** Yannick Joye, Jan Willem Bolderdijk

**Affiliations:** Department of Marketing, Faculty of Economics and Business, University of GroningenGroningen, Netherlands

**Keywords:** environmental psychology, extraordinary nature, awe-evoking natural environments, mundane natural environments, mood, awe, prosociality, social value orientation

## Abstract

Environmental psychology research has demonstrated that exposure to mundane natural environments can be psychologically beneficial, and can, for instance, improve individuals' mood and concentration. However, little research has yet examined the psychological benefits of extraordinary, awe-evoking kinds of nature, such as spectacular mountain scenes or impressive waterfalls. In this study, we aimed to address the underrepresentation of such extraordinary nature in research on human—nature interactions. Specifically, we examined whether watching a picture slideshow of awesome as opposed to mundane nature differentially affected individuals' emotions, mood, social value orientation (SVO), and their willingness to donate something to others. Our analyses revealed that, compared to mundane nature and a neutral condition, watching awesome natural scenes and phenomena had some unique and pronounced emotional effects (e.g., feeling small and humble), triggered the most mood improvement, and led to a more prosocial SVO. We found that participants' willingness to donate did not differ significantly for any of the conditions.

## Introduction

For many urbanites, spending time outdoors in nature can be a source of joy, relaxation, individual fulfillment, or even spiritual inspiration (Fredrickson and Anderson, 1999). In recent years, there has been a steady increase of psychological research into the beneficial psychological effects that can ensue from contact with natural as opposed to urban environments. Environmental psychology studies have shown, for example, that spending time in, or (passively) watching natural environments can bring about positive moods (Fredrickson and Levenson, 1998; Berman et al., 2008, 2012; Mayer et al., 2009; Ryan et al., 2010; Nisbet and Zelenski, 2011), replenish depleted attentional resources (Kaplan and Kaplan, 1989; Kaplan, 1995; Kaplan and Berman, 2010), and reduce stress (Ulrich et al., 1991). Based on these findings, having contact with natural environments and elements has been put forward as a therapeutic intervention (Maller et al., 2006) that can enhance concentration (Kaplan and Kaplan, 1989; Kaplan, 1995; Kaplan and Berman, 2010), can promote recovery from stress-related conditions (e.g., burnout, depression; Berman et al., 2012), or can make people feel generally more vital and happy (Ryan et al., 2010).

Most research on the beneficial effects of interactions with natural as opposed to urban environments is conducted in the context of what is known as “restorative environments research” (Hartig et al., 1996). Researchers in this field mainly investigate the ability of nature to recover or replenish depleted cognitive (Kaplan and Kaplan, 1989; Kaplan, 1995; Kaplan and Berman, 2010) or emotional resources (Ulrich et al., 1991). Of particular importance to the current paper is that the natural environments that are typically shown to respondents in restoration experiments—or the environments in which they are immersed—are often rather mundane natural settings. Examples are gardens, parks, waterfronts, or other types of nature, which are considered only to be “softly” fascinating (i.e., they moderately draw attention in a pleasant way; Herzog et al., 1997). The use of mundane nature as stimulus material is generally motivated by the fact that such nature produces little arousal, which might otherwise hamper the process of restoration (Kaplan and Kaplan, 1989; Kaplan, 1995; Kaplan and Berman, 2010).

Mundane natural settings, like parks and gardens, are probably among the most familiar and accessible types of nature for urban dwellers. People however also find great joy in experiencing extraordinary natural environments events or phenomena, such as the expansive view over the *Grand Canyon* or the splendid sight of the formidable *Victoria Waterfalls*, at the border of Zambia and Zimbabwe. The popularity of such awe-evoking nature scenes and phenomena as tourist destinations and as world heritage (e.g., Yellowstone National Park, USA) testifies to the emotional and/or aesthetic impact such nature can have on people.

Despite people's attraction to awesome nature, relatively little is known about the possible beneficial psychological and emotional effects of encounters with such extraordinary environments and phenomena (but see Cole and Hall, 2010). One source of knowledge is research into wilderness experiences (e.g., Talbot and Kaplan, 1986; Fredrickson and Anderson, 1999). This research has shown that wilderness settings can trigger a wide spectrum of emotions and states, ranging from feelings of mental refreshment and invigoration (Van den Berg and Ter Heijne, 2005), to intense fear, and even increased thoughts about death (Koole and Van den Berg, 2005). However, research into wilderness experiences is often qualitative and has not systematically focused on the psychological effects of encounters with the awe-evoking aspects of wilderness. Therefore, the general goal of the current study was to address this relative lack of (environmental psychology) attention to awesome nature by quantitatively exploring the distinct emotional and prosocial effects that might ensue from exposure to awesome as opposed to more mundane natural environments or phenomena.

According to Keltner and Haidt (2003) feelings of awe are triggered by vast and overwhelming phenomena, which defy an individual's understanding of the world, requiring an adaptive need for cognitive accommodation. Within the field of philosophical aesthetics (e.g., Burke, 1757/2008) and recent emotion research (Keltner and Haidt, 2003; Shiota et al., 2007) nature is often put forward as one of the most common sources of awe. However, not all types of nature elicit awe to the same extent. In Keltner and Haidt's (2003) view, awe is typically triggered by extraordinary nature scenes and elements, ranging from grand mountain scenery, to tornadoes, deep canyons, and lightning storms, and much less by more mundane types of nature, such as parks or gardens. With the current study we set out to explore the unique effects of extraordinary, awe-evoking nature on emotions, mood, and prosociality. We charted these effects by exposing participants to slideshows depicting either awesome nature, mundane nature (e.g., foliage, lawn), or pictures of neutral objects (e.g., a ladder; between-subjects design).

We had three particular aims with the current research. First, we aimed to explore the extent to which exposure to prototypical instances of awesome nature (e.g., high mountain peaks) would trigger awe, and awe-related emotions and states in individuals, as compared to more mundane types of nature and to a neutral condition. For the emotion measurement we found inspiration in the existing psychological literature on awe, which has characterized awe as a highly intense emotion, that is triggered by vast and overwhelming phenomena (Keltner and Haidt, 2003), leading to increased spirituality (e.g., Saroglou et al., 2008; Van Cappellen and Saroglou, 2012), and to feelings of oneness with, and caring for others (e.g., Shiota et al., 2007). Based on this characterization, we measured the extent to which the experience of awesome nature had been emotionally impactful, had triggered feelings of smallness and fear, and led to feelings of spirituality, care, and connectedness to others in participants. In addition to measuring these emotional states, we also probed how individuals had evaluated each of the slideshows. Based on the fact that awe is considered as an aesthetic emotion, triggered by information-rich and unexpected and extraordinary phenomena (Keltner and Haidt, 2003; Konecni, 2005), we asked participants to rate the beauty, interestingness, and surprising character of the slideshows.

Our second aim was to explore how exposure to awe-evoking nature would impact individuals' moods, and how such possible mood shifts differed from the mood effects caused by more mundane nature types and pictures of neutral things^1^. Research has demonstrated that watching nature can improve individuals' moods, both when they are stressed (Ulrich et al., 1991) and unstressed (Ryan et al., 2010). Less is however known about the positive effects of *awesome* nature on mood, and about how these (possible) mood effects may differ from the mood-enhancing capacities of more mundane natural environments and elements. In line with earlier research demonstrating the mood-enhancing effects of nature (e.g., Ulrich et al., 1991), we expected a (pre- to post-slideshow) mood improvement for individuals who had been watching a slideshow of mundane nature. Our expectations for those who had been watching awesome nature were mixed. Because awesome nature can sometimes have threatening aspects (e.g., tornadoes, thunderstorms; Keltner and Haidt, 2003), we deemed it conceivable that awesome nature would have a negative impact on participants' moods. However, inasmuch as the experience of awe has been characterized as a positive and uplifting emotion (Keltner and Haidt, 2003; Saroglou et al., 2008; Griskevicius et al., 2010), one could also expect a mood improvement after exposure to awesome nature. Another reason to think why awe-inspiring nature would have a positive impact on mood is that this type of nature potentially provides a higher or more intense dose of nature exposure compared to mundane nature.

The third aim of our study was to explore the possible effects of awesome vs. mundane nature on prosociality. This part of our research was inspired by the fact that exposure to mundane natural scenes/elements has been found to positively influence prosociality (Weinstein et al., 2009; Raihani and Bshary, 2012)^2^. A recent study by Guéguen and Stefan (2014), for example, showed that people displayed more helping behavior (i.e., picking up a glove off the ground and returning it to the stranger who dropped it) after immersion in an urban park than before entering the park. Quite similarly, Zhang et al. (2014) found that after exposure to beautiful nature, individuals behaved more prosocially than after exposure to less beautiful nature. Different studies have also shown that exposure to awe-evoking nature can trigger determinants of prosociality, such as general feelings of connectedness and oneness with others (Shiota et al., 2007; Saroglou et al., 2008; Rudd et al., 2012; Van Cappellen and Saroglou, 2012). While there is thus evidence that both mundane and awesome nature can positively impact prosociality, this effect has (mostly) been obtained by contrasting nature with non-natural environments (e.g., urban settings). This begs the question as to whether the finding of increased prosociality (or determinants of prosociality) merely reflects a generic nature effect, or whether some types of nature (especially awesome nature) have unique, and more pronounced positive effects on prosociality than mundane nature types. We aimed to address this issue in the current research by measuring participants' social value orientation (SVO) and their willingness to donate after exposure to either awesome nature, mundane nature, or the neutral condition.

As will be outlined below, the study consisted of five phases. First, participants were exposed to a slideshow depicting either awesome nature, mundane nature, or neutral pictures. Second, we measured the type and strength of the emotions which participants had experienced during each of the slideshows, as well as their evaluations of the slideshow pictures. Third—as an extension to the previous emotion measurement—we examined how watching the different slideshows had affected participants' moods. Fourth, we examined the influence of the different slideshow conditions on two prosociality measures: namely, participants' SVO and their willingness to donate. Finally, we examined whether awe mediated the effects of the environmental slideshows on mood improvement and prosociality measures.

## Methods

### Participants and design

This internet-based study was programmed in *Qualtrics*. Two-hundred and fifteen respondents participated in the study (129 females; *M*_*age*_ = 32.88, *SD* = 12.79), and they were recruited via the online crowdsourcing service *Amazon Mechanical Turk* from the US pool of respondents (Buhrmester et al., 2011). Each of them received 42 cents for participating. The study had a between-subjects design, with slideshow condition as the between-subjects variable. Average execution time for the experiment was approximately 17 min. Using the MAD outlier detection strategy by Leys et al. (2013) on total execution time, we excluded 13 respondents (6%) who had executed the experiment too slowly (i.e., more than 1454 s, or approximately 24 min)^3^.

## Materials

### Stimuli

**Figure 1:**
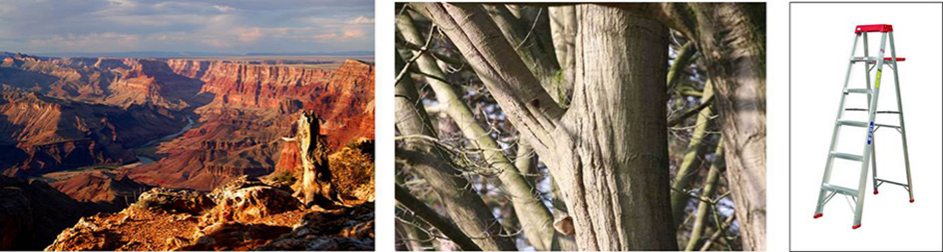
**Pictures of the awesome nature (left; credit: John Vetterli), mundane nature (middle), and neutral (right) condition**. Note that due to reasons of copyright, the image of awesome nature and the control condition are not the ones used in the actual study. They are however highly similar to the original images.

The stimuli of this study were three different slideshows, each consisting of 14 different color photographs. Image resolution was kept relatively low (i.e., maximum 800 by 800 pixels) to ensure that the entire image would be fully displayed on the (computer) screen of all participants. The three slideshows depicted either neutral pictures (i.e., the “neutral” condition) or pictures of one of two nature types, namely “awesome nature” and “mundane nature” (see Figure 1 for sample pictures of these two nature conditions and the neutral condition)^4^. Using photographs of environments as experimental stimuli is highly common in environmental psychology research.

The pictures of the awesome nature condition and neutral condition were collected from the internet by the authors, whereas the photographs from the mundane nature condition had been taken by one of the authors. The awesome nature condition consisted mainly of pictures of grand and dramatic mountain scenes, but we also included some pictures of grand landscapes dominated by a thunderstorm, tornado, rainbow, and sunsets. Our choice for these particular landscape types and/or natural phenomena was based on a close reading of the literature on awe, in which it is often suggested that these landscape types and natural phenomena are amongst the most common and powerful elicitors of awe (Keltner and Haidt, 2003). The mundane nature condition consisted of photographs of everyday natural elements, such as grass, foliage, or trees, and small-scale natural scenes dominated by vegetative elements. All sites and elements of the mundane nature condition were photographed by the first author in Belgium during autumn. By focusing on relatively small scenes and individual elements we made sure that this nature condition was lacking grandeur and was devoid of any “powerful” natural elements which might trigger awe. The neutral condition consisted of pictures of everyday objects, such as a ladder, bucket, or a chair, which—we assumed—would leave the respondents largely emotionally unaffected.

#### Measures

***Mood measurements***. We took two mood measurements. For each of these measurements, participants had to indicate the mood they were in at that moment, using a sliding scale ranging from 0 (the worst ever) to 100 (the best ever; scale taken from Sherman et al., 2009).

***Emotion measurement***. We asked participants how “awed,” “fearful,” “spiritual,” “caring,” and “connected to others” they had felt while watching the slideshow (scored from 1 [not at all] to 7 [very much]). We also measured how much the slideshow had emotionally affected them (sliding scale from 0 [not at all] to 100 [very much]; scale taken from Sherman et al., 2009), and how much they had experienced feelings of “smallness,” “humility,” and “respect” (scored from 1 [not at all] to 7 [very much]). For the latter three items we created a smallness index (Cronbach's α = 0.83). Note that we made this selection of individual emotion items, based on a reading of the psychological literature on awe (e.g., Shiota et al., 2007; Saroglou et al., 2008), and on the broader emotion research literature.

***Slideshow evaluations***. In addition to probing participants' emotions, we also asked them to evaluate the slideshow they had seen. We specifically asked them how “entertaining” and “interesting” they found the slideshow pictures (scored from 1 [not at all] to 7 [very much]) and we created an “interest” index by averaging both items (Cronbach's α = 0.83). We also measured how “surprising” and “beautiful” they found the slideshow (scored from 1 [not at all] to 7 [very much]; items taken from Sherman et al., 2009).

***Willingness to donate***. Willingness to donate was measured by asking participants to indicate to what extent they would be willing to help the victims of a disaster (i.e., a flooding or an earthquake) by donating the following items: food, clothing, money, and blood^5^. The scale to measure willingness to donate/help was developed by ourselves, and consisted of a sequence of nine progressively longer arrow-bars. The shortest (coded as 1) and longest arrow-bar (coded as 9) represented respectively the lowest and the highest levels of donating/helping (see Figure A1 in the Supplementary Material for the picture). A donation index was calculated by averaging the scores of the four donation scores (Cronbach's α = 0.91).

***Social value orientation***. SVO was measured by asking participants to make a choice that involved outcomes for themselves and another (imaginary) person (scale taken from Van Lange et al., 1997). Specifically, participants were given three possible point distributions, and they had to indicate which choice distribution they preferred. For each choice, they could choose between a prosocial (e.g., You get: 480—Other gets: 480), individualistic (e.g., You get: 540—Other gets: 280), or a competitive (e.g., You get: 480—Other gets: 80) choice distribution. Participants had to make a total of nine choices. For each choice we calculated an SVO score by dividing the points attributed to the “Other” by the total amount of points that had been distributed in that choice (Bekkers, 2004). For example, the choice for the distribution “You get: 480—Other gets: 480” led to an SVO score of 0.50 (i.e., 480/[480 + 480]), whereas the choice for the distribution “You get: 480—Other gets: 80” corresponded to an SVO score of 0.14 (i.e., 80/[480 + 80]). We calculated an overall SVO score by averaging the SVO scores for all nine choices presented to the participants, with the highest scores representing the most prosocial SVO orientation (Cronbach's α = 0.97). Note that although SVO is typically conceptualized as an individual difference, research also indicates that it can be affected by contextual factors (De Dreu and McCusker, 1997) and even by mood and emotions (cfr., Hertel et al., 2000; Ketelaar and Au, 2003).

### Procedure

The study began by asking participants' for their age and gender. Directly after this, we conducted a first mood measurement. Participants were then randomly assigned to either the awesome nature (*n* = 70), mundane nature (*n* = 68), or neutral (*n* = 64) slideshow condition. We asked them to watch each slideshow picture for at least 10 s before proceeding to the next picture. On average, participants clicked away after 12 s (*M* = 12.03, *SD* = 5.78), suggesting a very reasonable viewing time per picture. A One-Way ANOVA showed that viewing time per picture did not differ significantly across the three conditions, *F*_(2, 199)_ = 0.65, *p* = 0.521. Directly after the slideshow, we took the emotion measurement and slideshow evaluation, which were directly followed by the second, post-slideshow mood measurement. In the following phase we measured participants' willingness to donate and their SVO. At the end of the study participants had the opportunity to comment on the study (none of the participants guessed the purpose of the study).

## Results

### Effects on emotions and slideshow evaluations

We conducted a One-Way ANOVA with slideshow condition (i.e., awesome, mundane, and neutral) as the between-subjects factor, and the emotions and slideshow evaluations as the dependent variables. All analyses showed that there were statistically significant differences between the three conditions. We discuss the most relevant findings here. All descriptive statistics, *F*-statistics and contrasts per condition can be found in Table 1.

**Table 1:** **Means (standard deviations) per condition for scores on the emotion items and slideshow evaluations, and overall *F*-statistics**.

	**Awesome**	**Mundane**	**Neutral**	***F***	***p***	**η^2^_*p*_**
Awe	6.21 (0.97)_a_	4.34 (1.50)_b_	2.16 (1.25)_c_	173.71	<0.001	0.63
Emotionally affected	51.91 (26.98)_a_	39.44 (30.90)_b_	16.73 (22.36)_c_	28.82	<0.001	0.22
Smallness	5.53 (1.20)_a_	4.17 (1.03)_b_	2.76 (1.43)_c_	84.94	<0.001	0.46
Fear	3.21 (1.89)_a_	1.84 (1.31)_b_	1.94 (1.44)_b_	16.28	<0.001	0.14
Spiritual	5.01 (2.01)_a_	4.62 (1.52)_a_	2.67 (1.67)_b_	33.58	<0.001	0.25
Care	4.36 (1.50)_a_	4.76 (1.30)_a_	3.09 (1.72)_b_	21.62	<0.001	0.17
Connectedness	4.03 (1.61)_a_	3.79 (1.60)_a_	2.94 (1.84)_b_	7.63	=0.001	0.07
Beauty	6.61 (0.68)_a_	5.91 (1.14)_b_	3.20 (1.56)_c_	155.10	<0.001	0.60
Interest	5.96 (0.94)_a_	5.02 (1.29)_b_	3.60 (1.70)_c_	52.15	<0.001	0.34
Surprise	5.20 (1.46)_a_	3.34 (1.61)_b_	3.20 (1.67)_b_	34.01	<0.001	0.25

Means not sharing subscripts differ significantly at p < 0.05 according to Tukey's Honestly Significant Difference comparison.

We found that participants who had watched the slideshow of awesome nature had indeed experienced significantly more awe than participants who had watched the mundane nature or neutral condition, thus providing a manipulation check of our stimulus set. As expected, the slideshow of awesome nature was considered as significantly more beautiful than the mundane nature and neutral condition. In the awesome nature condition participants felt significantly smaller (as measured by the smallness index) than in the mundane nature and neutral conditions, which is consistent with Keltner and Haidt's (2003) claim that awe-evoking stimuli are often characterized by a vastness that can dwarf the spectator. Awe-evoking nature also elicited significantly more fear than the other two conditions. Of all conditions, the awesome nature condition affected the participants emotionally the most, and sparked significantly higher levels of interest (cfr., the interest index) and surprise than the other two conditions. Finally, in both nature conditions participants indicated feeling significantly more connected to others, more caring, and more spiritual than in the neutral condition. There were however no significant differences between the two nature conditions on these last three items.

### Effects on mood

We used a mixed design ANOVA to examine the influence of the different slideshow conditions on the evolution of participants' mood. Time of mood measurement (i.e., pre- or post-slideshow) was entered as the within-subjects variable, slideshow condition as the between-subjects variable, and mood score as the dependent variable. Means and standard deviations of pre- and post-slideshow mood scores are presented in Table 2. Mauchly's test of sphericity was significant, so Huynh–Feldt tests were used. There was a main effect of time of mood measurement, *F*_(1, 199)_ = 19.24, *p* < 0.001, η^2^_*p*_ = 0.08, showing that an overall pre- to post-slideshow mood improvement had occurred.

**Table 2:** **Means (standard deviations) per condition for pre- and post-slideshow mood measurement scores and for mood improvement**.

	**Awesome**	**Mundane**	**Neutral**
Pre-slideshow mood	61.90 (16.64)_a_	66.91 (17.82)_a_	61.23 (17.48)_a_
Post-slideshow mood	70.85 (15.57)_a_	70.04 (15.96)_a_	59.26 (17.48)_b_
Mood improvement	8.95 (10.60)_a_	3.13 (11.05)_b_	–1.96 (11.12)_c_

Means not sharing subscripts differ significantly at p < 0.05 according to Tukey's Honestly Significant Difference comparison. Subscripts compare the means within each row.

**Figure 2:**
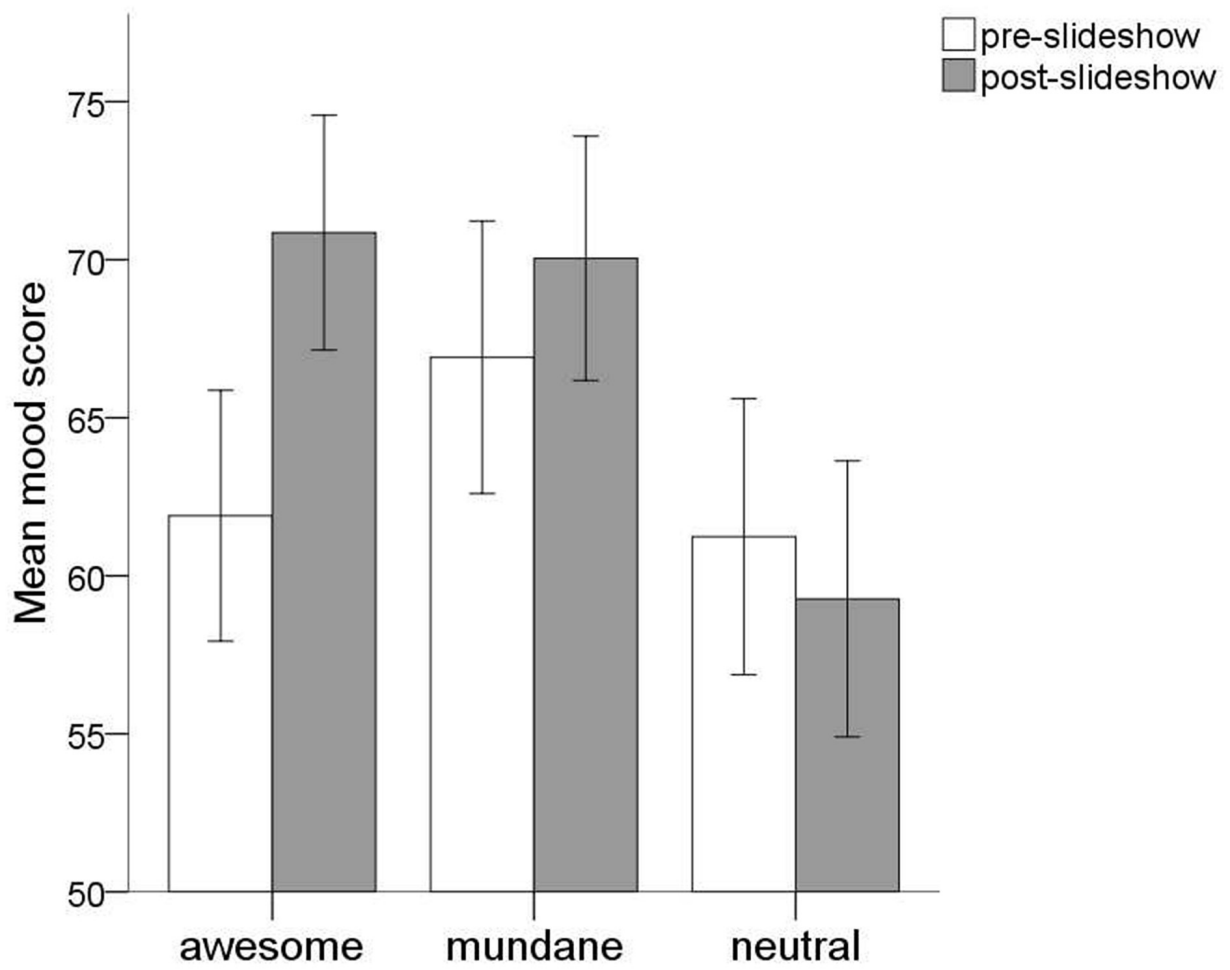
**Pre- and post-slideshow mood scores as a function of (slideshow) condition (error bars represent 95% confidence intervals)**.

There was also a significant time of mood measurement by slideshow condition interaction, *F*_(2, 199)_ = 16.79, *p* < 0.001, η^2^_*p*_ = 0.14, indicating that mood changes differed depending on slideshow condition (see Figure 2). Specifically, in the two nature conditions, there was a pre- to post-slideshow mood improvement [awesome nature: *F*_(1, 69)_ = 49.95, *p* < 0.001, η^2^_*p*_ = 0.42; mundane nature: *F*_(1, 67)_ = 5.46, *p* = 0.022, η^2^_*p*_ = 0.07] whereas mood scores stayed virtually the same over time in the neutral condition [*F*_(1, 63)_ = 2.00, *p* = 0.162, η^2^_*p*_ = 0.03]. A significant time of mood measurement by slideshow condition interaction still remained, when only the two nature conditions were considered *F*_(1, 136)_ = 9.98, *p* = 0.002, η^2^_*p*_ = 0.06. This interaction shows a steeper pre- to post-slideshow mood improvement in the awesome nature condition than in the mundane nature condition.

To get a clearer picture of how mood changes differed across the three slideshow conditions, we calculated the pre- to post-slideshow difference in mood scores. A One-Way ANOVA with mood improvement as dependent variable and slideshow condition as between-subjects factor revealed statistically significant differences between the three conditions, *F*_(2, 199)_ = 16.79, *p* < 0.001, η^2^_*p*_ = 0.14. *Post-hoc* comparisons indicated that mood improvement scores in the mundane nature condition differed significantly from those in the neutral condition, thus corroborating earlier research that also everyday types of nature can uplift moods (Ryan et al., 2010). Importantly, we also found that exposure to awesome nature improved moods above and beyond the effect of mundane nature condition, testifying to the unique mood-enhancing effects of the former nature type^6^. Means and standard deviations of mood improvement scores are presented in Table 2.

### Effects on willingness to donate and SVO

**Figure 3:**
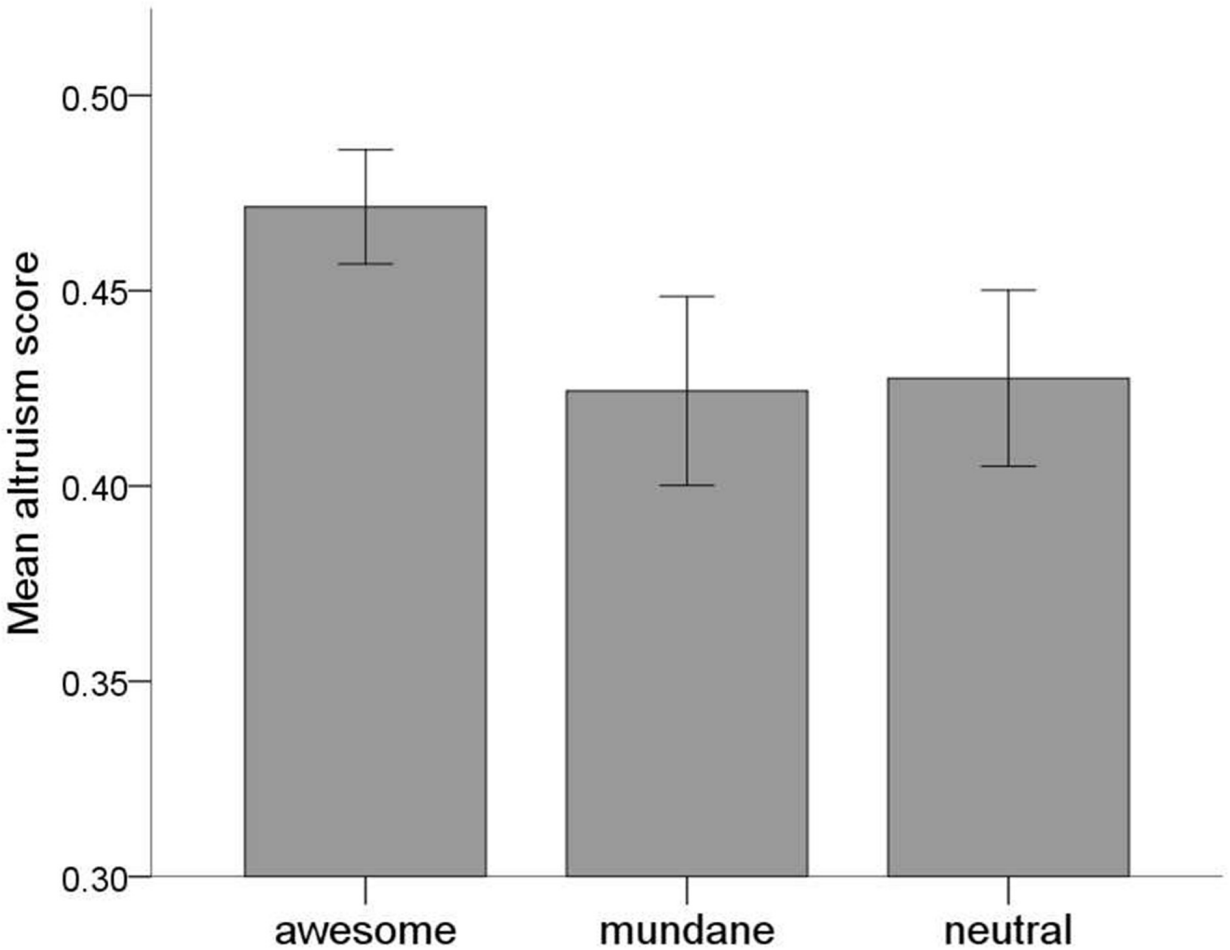
**Scores on the overall SVO score as a function of (slideshow) condition (error bars represent 95% confidence intervals)**.

A One-Way ANOVA with the donation index as dependent variable and slideshow condition as the between-subjects factor showed that slideshow condition did not have a significant influence on willingness to donate/help, *F*_(2, 199)_ = 1.05, *p* = 0.351, η^2^_*p*_ = 0.01 (see Table 3 for means and standard deviations)^7^. In contrast, a One-Way ANOVA with the overall SVO score as the dependent variable and slideshow condition as the between-subjects factor showed that slideshow condition had a significant impact on the overall SVO score, *F*_(2, 199)_ = 6.57, *p* = 0.002, η^2^_*p*_ = 0.06 (see Table 3 for means and standard deviations). While the overall SVO score was generally high, *post-hoc* comparisons showed that in the awesome nature condition participants made significantly more prosocial choices than in the mundane nature and neutral condition. There were no significant differences on the overall SVO score between the mundane nature and neutral condition (see Figure 3). Note also that there was no significant correlation between the overall SVO score and the donation index, *r*_(200)_ = 0.05, *p* = 0.479.

**Table 3:** **Means (standard deviations) per condition for donations and social value orientation**.

	**Awesome**	**Mundane**	**Neutral**
Donation	6.38 (1.81)_a_	6.77 (1.92)_a_	6.77 (1.78)_a_
Social value orientation	0.47 (0.06)_a_	0.42 (0.09)_b_	0.42 (0.09)_b_

Means not sharing subscripts differ significantly at p < 0.05 according to Tukey's Honestly Significant Difference comparison.

### Mediation analyses

Based on theory and research suggesting that the emotion of awe can (a) uplift moods and (b) can lead to increased prosociality (Keltner and Haidt, 2003), we tested whether the difference between the two nature conditions on mood improvement scores and on the overall SVO score was mediated by experienced awe. All conditions for mediation were met: (a) awesome nature led to higher levels of awe than mundane nature (as discussed above); (b) awesome nature was more mood improving and led to higher overall SVO scores than the mundane nature condition (as discussed above); and (c) there was a positive and significant correlation between awe and mood improvement, *r*_(136)_ = 0.32, *p* < 0.001, and between awe and the overall SVO score, *r*_(136)_ = 0.27, *p* = 0.001.

**Figure 4:**
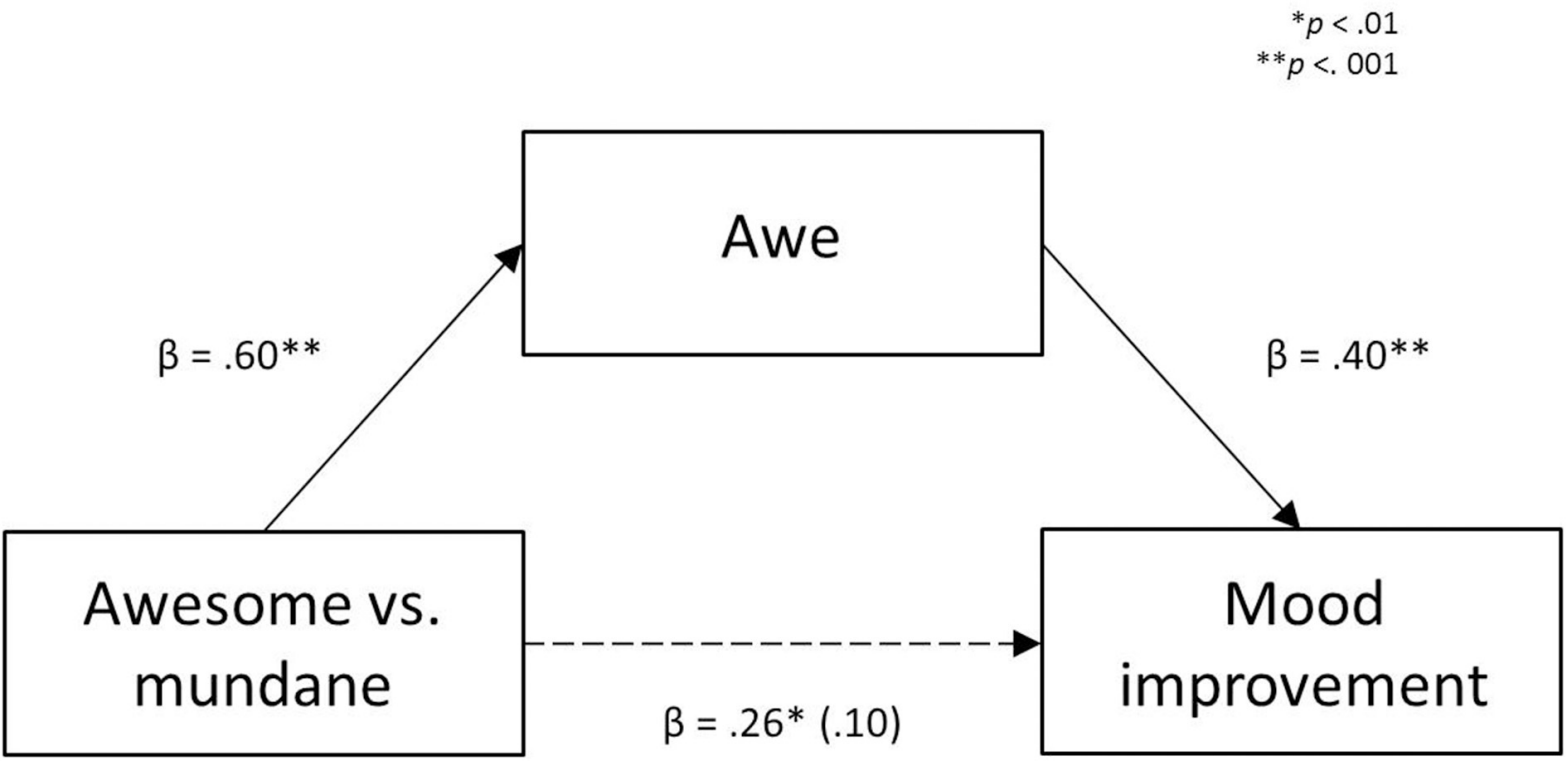
**The effect of awesome vs. mundane nature on mood is mediated by awe**. Note that we obtained the beta coefficients by running separate regressions in which we directly compared awesome (dummy-coded as 1) with mundane nature (dummy-coded as 0).

We made use of Preacher and Hayes' bootstrap method for testing mediation, employing the SPSS macro *PROCESS* (Model 4) developed by Hayes (2013). We first tested whether awe could account for the documented differences in mood. We entered mood improvement scores as the dependent variable, slideshow condition (awesome vs. mundane) as the independent variable, and awe as the proposed mediator (See Figure 4 for a graphical depiction). The analysis showed that the bias-corrected 95% confidence interval (1000 bootstrap samples) for the indirect effect of slideshow condition (mundane vs. awesome) through awe did not include zero (−7.79 to −0.53). This is consistent with the interpretation that the mood-lifting effect of awesome nature (as compared to mundane nature) indeed stemmed from the feeling of awe which participants had experienced during the slideshow. We then tested whether awe could account for the documented differences in SVO (as discussed above). We conducted a second mediation analysis, now with the overall SVO score as the dependent variable. A bias-corrected 95% confidence interval (1000 bootstrap samples) for the indirect effect of slideshow condition (awesome vs. mundane) through awe included zero (−0.05 to 0.00). So, we could not confirm that the differential effect of the nature conditions (awesome vs. mundane nature) on the overall SVO score was caused by awe.

Although we were primarily interested in the unique effects of awesome nature (as opposed to more mundane nature), we also found that exposure to mundane nature led to significantly higher levels of awe, as well as to significantly better moods than the neutral condition. We therefore also explored the mediating role of awe in explaining the differences in mood improvement between the neutral and mundane nature conditions. We ran a mediation analysis with mood improvement scores as the dependent variable, slideshow condition (mundane vs. neutral) as the independent variable, and awe as the proposed mediator. Because a bias-corrected 95% confidence interval (1000 bootstrap samples) for the indirect effect of slideshow condition (mundane vs. neutral) through awe did include zero (−7.50 to 0.31), we were unable to conclude that that the mood lifting effect of mundane nature was caused by awe.

## Discussion

In most research on human—nature interactions there has been a tendency to investigate the beneficial psychological effects of fairly mundane natural landscapes on individuals (e.g., parks, garden, forests), with relatively little attention to the potentially beneficial psychological effects of exposure to more extraordinary kinds of natural environments and/or phenomena. With the current exploratory study we wanted to test whether landscapes and natural phenomena that are commonly considered as typical elicitors of the emotion awe—for example, soaring mountain peaks or spectacular waterfalls—would have unique effects on emotions, moods, and prosociality, as compared to more mundane types of nature and a neutral condition.

We began our study by exploring how participants evaluated, and emotionally responded to a slideshow consisting of pictures of either awesome or mundane nature, or to a slideshow with images of neutral objects. Our general finding was that watching photographs of awesome nature had unique emotional effects. Specifically, our analyses confirmed the assumption that awe is most typically triggered by grand natural landscapes and phenomena, such as impressive mountain scenery, tornadoes, or thunderstorms (Keltner and Haidt, 2003). We also found that awesome nature made respondents feel the smallest of all three slideshow conditions, thus supporting the claim that awe is a an emotional response to exceptional (natural) vastness (Keltner and Haidt, 2003).

Further analyses revealed that, from the two nature conditions, the images of extraordinary nature were considered as the most surprising, interesting, emotionally the most intense, and most beautiful. These results are consistent with the view that awesome nature is often very uncommon and/or contains unexpected phenomena, leading to an attentionally and emotionally gripping experience (Keltner and Haidt, 2003). While past research has shown that awe can bring individuals into a spiritual mindset (Saroglou et al., 2008) and lead to feelings of connectedness to others (Van Cappellen and Saroglou, 2012), we found no differences between the two nature conditions on feelings of care, connectedness to others, or spirituality. There were however statistically significant differences between the two nature conditions and the neutral condition on these three measures.

In the second phase of our study, we looked at whether the three slideshow conditions differentially affected pre- to post-slideshow changes in (self-reported) mood. In spite of the fact that the awesome nature condition was causing the highest amount of fear in participants, our analyses revealed that this type of nature also improved moods significantly more than the mundane nature condition (note however that mood was similar after exposure for both nature groups). It thus appears that not only mundane natural environments, which have received most attention in research on human—nature interactions, but also natural scenes and phenomena with somewhat threatening and fearful characteristics, can have invigorating effects.

In the third phase of the study, we explored the extent to which attending to awesome nature impacted prosociality. While the results from the emotion measurements did not reveal any statistically significant differences between the two nature conditions on proxies of prosociality (i.e., caring and connectedness to others), our analyses showed that after having watched the awesome nature slideshow, respondents became somewhat more prosocial (in terms of SVO) than those in the mundane nature and neutral condition. This result not only suggests that SVO is malleable by contextual, i.e., environmental factors, but also that some particular types of nature can make people more other-oriented than other types of nature. The fact that we did not find any effect of our manipulation on willingness to donate is inconsistent with earlier research showing that SVO translates into higher donations (Van Lange et al., 2007). A plausible explanation for the null-effect on donations is that participants were possibly confused by the unconventional scale we used for measuring willingness to donate (i.e., arrow-bars).

The last phase of our study consisted of exploring the possible mechanisms underlying the influence of awesome nature on mood improvement and SVO. We were unable to confirm that awe mediated the effect of slideshow condition on the overall SVO score. However, in agreement with the idea that experiencing awesome phenomena can be emotionally uplifting, a mediation analysis revealed that awe mediated the relationship between the two different nature conditions and mood improvement. Whereas research has suggested that (mundane) nature's mood-enhancing effect can be driven by generalized positive affect (Ulrich et al., 1991), the current research reveals that for awe-evoking nature there is a complementary pathway through which mood improvements can occur: namely by experiencing the particular emotion of awe.

There are, of course, a number of limitations to the current exploratory study. A first one is that the awesome nature condition consisted of a mix of different kinds of awe-evoking scenes and phenomena, making it impossible for us to say which pictures within the awesome nature condition had the strongest effects on our dependent variables. This issue could be resolved by using a more homogenous set of pictures in future studies (e.g., only pictures of mountain scenes). Second, the exposure to the slideshow pictures was relatively brief and virtual. Future research should therefore investigate which effects would occur with repeated/chronic exposure to actual awesome vs. mundane natural environments, and how long the effects (if any) persist after the awe-evoking stimulus has been taken away. A third limitation is that we have probed for awe and awe-related emotions by using only one item. This underscores the need to develop a validated scale—or any other measurement instrument—that is capable of accurately capturing the occurrence of this complex emotional state, and of associated emotions and states. Fourth, because the study was exploratory, we assessed mood using self-report measures. We hope that in future studies, more objective and validated measures will be used to gauge the positive psychological impact of awesome nature, and that, in addition to mood and emotion effects, also (possible) restorative effects will be investigated. Fifth, by having selected only US participants, our population sample was narrow and replicating these findings with participants from different cultures is therefore desirable. Finally, a well-known drawback of statistical mediation is that we cannot say anything about the direction of the effect. While in our interpretation experiencing awe caused the mood improvement, it cannot be excluded that it was actually the experienced mood improvement which caused people to experience higher levels of awe.

In conclusion, this exploratory paper provided evidence that the relative lack of attention to the positive behavioral and psychological effects of viewing extraordinary, awe-evoking natural environments is unjustified. Our general finding, and the message we want to convey is that awesome nature appears to have some very distinct (positive) effects on moods, emotions, and prosociality (e.g., highest SVO scores), which diverge from the effects obtained for more mundane types of nature. Regarding interventions, it is obviously far from evident to bring people into contact with actual awesome nature on a regular basis (because of its uncommon and often inaccessible character). However, as our results show, already brief exposure to relatively small images of awesome nature (under the form of, say, landscape posters or screensavers) may have significant positive effects on people's emotions and behavior.

## Author contributions

Yannick Joye and Jan Willem Bolderdijk conceived and designed the study. Yannick Joye analyzed the results and wrote an initial draft based on these results. Jan Willem Bolderdijk critically revised the draft manuscript and made important changes in content. Both authors of the paper agree to be accountable for the accuracy and integrity of any part of the work.

### Conflict of interest statement

The authors declare that the research was conducted in the absence of any commercial or financial relationships that could be construed as a potential conflict of interest.

## Footnotes

^1^One of the core differences between moods and emotions is that emotions last longer than emotions, they are more diffuse, and more global. Whereas emotions are mostly elicited by a temporally near stimulus, moods can be temporally remote from their cause and this cause may not always be easily identifiable.

^2^Possible reasons why nature exposure might be linked to increases in prosociality is that nature can lead to positive affect (Ulrich et al., 1991) and recover from ego depletion (Kaplan and Berman, 2010), both of which are shown to be linked to increases in prosociality (e.g., George, 1991; Xu et al., 2012).

^3^Including these outliers did not substantially alter the results.

^4^Note that we also included a fourth condition consisting of pictures of tiny natural elements (e.g., a tiny flower). We decided to drop this condition from the analyses, because—in hindsight—we realized that tiny natural elements might have been perceived as cute. As cute things are known to have particular effects on one's moods, emotions and prosocial tendencies (Sherman and Haidt, 2011), we deemed it inappropriate to pit the emotional and prosocial effects of awesome nature against this type of nature. The main results for the tiny nature condition can be found in Table A1 in the Supplementary Material.

^5^We also asked participants how much they would be willing to pray for the victims. We decided to drop this item from our analyses because praying does not refer to something material like the other items, making it a far less suitable item to capture participants' willingness to donate.

^6^Note that there were differences in mood (albeit non-significant) between some of the conditions before slideshow exposure, i.e., participants in the mundane nature condition were in a better mood compared to the other two conditions. We therefore conducted an ANCOVA with post-slideshow mood as the dependent variable, slideshow condition as the independent variable, and pre-slideshow mood as the covariate. This analysis revealed a significant main effect of slideshow condition on post-slideshow mood, *F*_(2, 198)_ = 20.60, *p* < 0.001, η^2^_*p*_ = 0.17, and showed a positive and significant effect of the covariate pre-slideshow mood, *F*_(1, 198)_ = 330.39, *p* < 0.001, η^2^_*p*_ = 0.62. Contrast analyses showed that post-slideshow mood levels were significantly higher in the awesome nature than in the mundane nature and neutral condition, and that participants' post-slideshow moods were significantly better in the mundane nature than in the neutral condition.

^7^Willingness to pray was not significantly affected by the three slideshow conditions, *F*_(2, 199)_ = 0.85, *p* = 0.429, η^2^_*p*_ = 0.00.
