# Supplementary material for: An exploratory study into the effects of extraordinary nature on emotions, mood, and prosociality
Source: Front Psychol. 2015 Jan 28;5:1577. doi: 10.3389/fpsyg.2014.01577 (PMC4309161; doi:10.3389/fpsyg.2014.01577)
Supplement: Supplementary file 1 [file DataSheet1.DOCX]

**Appendix**

**Figure A1**. Sequence of arrow-bars used to measure participants’ willingness to donate.


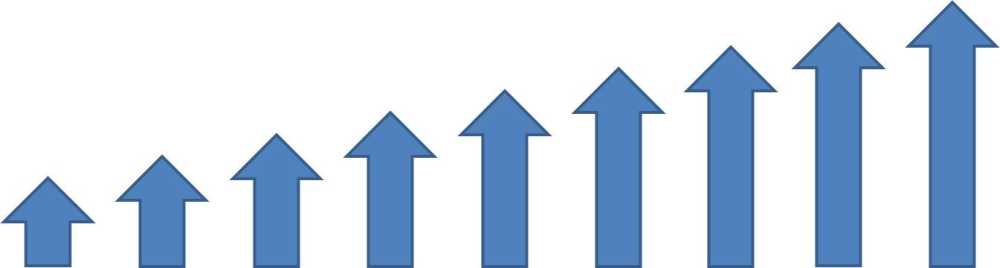


**Table A1.** Means (standard deviations) of the awesome nature, mundane nature, tiny nature and neutral condition for the main dependent measures discussed in the paper.

|  | Awesome | Mundane | Neutral | Tiny | *F* | *p* | $\boldsymbol{\eta}_{\boldsymbol{p}}^{\boldsymbol{2}}$ |
| --- | --- | --- | --- | --- | --- | --- | --- |
| Awe | 6.21 (0.97)_a_ | 4.34 (1.50)_b_ | 2.16 (1.25)_c_ | 5.08 (1.19)_d_ | 125.00 | < .001 | 0.59 |
| Emotionally affected | 51.91 (26.98)_a_ | 39.44 (30.90)_b_ | 16.73 (22.36)_c_ | 44.03 (25.29)_ab_ | 21.04 | < .001 | 0.19 |
| Smallness | 5.53 (1.20)_a_ | 4.17 (1.03)_b_ | 2.76 (1.43)_c_ | 4.18 (1.06)_b_ | 60.12 | < .001 | 0.41 |
| Fear | 3.21 (1.89)_a_ | 1.84 (1.31)_b_ | 1.94 (1.44)_b_ | 1.42 (0.86)_b_ | 19.54 | < .001 | 0.18 |
| Spiritual | 5.01 (2.01)_a_ | 4.62 (1.52)_ac_ | 2.67 (1.67)_b_ | 4.16 (1.58)_c_ | 23.43 | < .001 | 0.21 |
| Care | 4.36 (1.50)_a_ | 4.76 (1.30)_ac_ | 3.09 (1.72)_b_ | 5.16 (1.29)_c_ | 23.75 | < .001 | 0.21 |
| Connectedness | 4.03 (1.61)_a_ | 3.79 (1.60)_a_ | 2.94 (1.84)_b_ | 3.89 (1.38)_a_ | 6.00 | .001 | 0.06 |
| Beauty | 6.61 (0.68)_a_ | 5.91 (1.14)_b_ | 3.20 (1.56)_c_ | 6.29 (0.89)_ab_ | 127.26 | < .001 | 0.59 |
| Interest | 5.96 (0.94)_a_ | 5.02 (1.29)_b_ | 3.60 (1.70)_c_ | 5.36 (1.05)_b_ | 40.20 | < .001 | 0.31 |
| Surprise | 5.20 (1.46)_a_ | 3.34 (1.61)_b_ | 3.20 (1.67)_b_ | 4.26 (1.37)_c_ | 24.71 | < .001 | 0.22 |
| Mood improvement | 8.95 (10.60)_a_ | 3.13 (11.05)_b_ | -1.96 (11.12)_c_ | 6.37 (8.34)_ab_ | 13.58 | < .001 | 0.13 |
| Donation | 6.38 (1.81)_a_ | 6.77 (1.92)_a_ | 6.77 (1.78)_a_ | 6.22 (2.15)_a_ | 1.37 | = .25 | 0.01 |
| Social value orientation | 0.47 (0.06)_a_ | 0.42 (0.09)_b_ | 0.42 (0.09)_b_ | 0.45 (0.06)_ab_ | 5.21 | = .002 | 0.05 |
| *Note.* Means not sharing subscripts differ significantly at p <.05 according to Tukey’s Honestly Significant Difference comparison. | | | |  |  |  |  |
